# Cocaine

**Published:** 1885-01

**Authors:** 


### Cocaine.

The Vienna correspondent of the Philadelphia *Medical News* says of the new local anesthetic: There is no doubt about the fact that the medical virtues of erythroxylon coca are much better known and appreciated in America than in Europe. But recently, since E. Merck, in Darmstadt, has begun to prepare the alkaloid of that plant, and sell the easily soluble combination of cocaine with the hydrochloric acid—though for a remarkably high price—some use has been made of cocaine, and some experience has been collected in Vienna about the interesting drug. Prof. Fleische and his colleagues here have seen the excellent effect of cocaine during the period of "abstinentia morphiæ." Persons used to large amounts of morphia for many years could bear the privation of this alkaloid without suffering the well-known tortures which are usually connected with it. Even in cases in which the morphia was not withdrawn gradually, but stopped at once, cocaine showed the best effects.

But we do not propose to dwell on those effects of cocaine which are already known, but about a form of application which, so far as is known, is quite new. This application has been brought forth by Dr. Koller, in Vienna. Starting from the fact that the parts of the tongue which were in direct contact with a strong solution of cocaine lose, for a certain time, their sensibility, he was led to try the application of cocaine to the cornea. If one or two drops of a concentrated solution of cocaine hydrochlorate in water be applied for some minutes to the free surface of the eye, the sensitive nerves of the cornea and of the surrounding parts become paralyzed, there is local anesthesia, and the operations of extraction of cataract, of iridectomy, etc., can be performed without giving the patient any pain.

The great value of cocaine, as a means of inducing local anesthesia in the eye, is evident.

There have been performed also some operations (here in Vienna) on the larynx, in which a complete local anesthesia was effected by cocaine, but in that way it has been used previously, we are informed.

